# Inhibition of autophagy sensitizes malignant pleural mesothelioma cells to dual PI3K/mTOR inhibitors

**DOI:** 10.1038/cddis.2015.124

**Published:** 2015-05-07

**Authors:** N Echeverry, G Ziltener, D Barbone, W Weder, R A Stahel, V C Broaddus, E Felley-Bosco

**Affiliations:** 1 Laboratory of Molecular Oncology, Clinic of Oncology, University Hospital of Zürich, Häldeliweg 4, Zürich 8044, Switzerland; 2 Department of Medicine, Division of Pulmonary, San Francisco General Hospital, University of California San Francisco, San Francisco, CA 94110, USA; 3 Division of Thoracic Surgery, University Hospital Zürich, Zürich, Switzerland

## Abstract

Malignant pleural mesothelioma (MPM) originates in most of the cases from chronic inflammation of the mesothelium due to exposure to asbestos fibers. Given the limited effect of chemotherapy, a big effort is being made to find new treatment options. The PI3K/mTOR pathway was reported to be upregulated in MPM. We tested the cell growth inhibition properties of two dual PI3K/mTOR inhibitors NVP-BEZ235 and GDC-0980 on 19 MPM cell lines. We could identify resistant and sensitive lines; however, there was no correlation to the downregulation of PI3K/mTOR activity markers. As a result of mTOR inhibition, both drugs efficiently induced long-term autophagy but not cell death. Autophagy blockade by chloroquine in combination with the dual PI3K/mTOR inhibitors significantly induced caspase-independent cell death involving RIP1 in the sensitive cell line SPC212. Cell death in the resistant cell line Mero-82 was less pronounced, and it was not induced via RIP1-dependent mechanism, suggesting the involvement of RIP1 downstream effectors. Cell death induction was confirmed in 3D systems. Based on these results, we identify autophagy as one of the main mechanisms of cell death resistance against dual PI3K/mTOR inhibitors in MPM. As PI3K/mTOR inhibitors are under investigation in clinical trials, these results may help interpreting their outcome and suggest ways for intervention.

Malignant pleural mesothelioma (MPM) is sensitive to phosphatidylinositol 3-kinase/mammalian target of rapamycin (PI3K/mTOR) signaling inhibitors due to the activation of PI3K/mTOR signaling.^1, 2^ The activation may result from inactivation of INP4A phosphatase, which is downregulated in 44% of MPM (presented at IMIG2014), or alterations in PI3K signaling components, which are mutated in 9% of MPM,^3^ while receptor tyrosine kinase mutations/amplifications have not been identified in two recent high-throughput studies.^4, 5^

One of the tumor-suppressor genes frequently mutated in MPM is NF2 and NF2-null cells were shown to be sensitive to growth-inhibitory effects of rapamycin^6^ via mechanisms involving PI3K signaling-independent mTORC1 activation. However, the mTOR inhibitor, everolimus, showed no therapeutic benefit in unselected MPM patients.^7^ As mTORC1 inhibitors often lead to a feedback activation of PI3K activation in cancers,^8, 9^ we postulated that dual PI3K–mTOR inhibitors may yield greater therapeutic benefit. Furthermore, NF2 was also shown to inhibit PI3K activity by binding to PI3K enhancer-L (PIKE-L), which disrupts binding of PIKE-L to PI3K^10^ and loss of NF2 in schwannoma was shown to sensitize to PI3K inhibitors.^11^

In a screen on the dual PI3K/mTOR inhibitor NVP-BEZ235, within the Sanger Institute/MGH's ‘Genomics of Drug Sensitivity' screening panel,^12^ CDKN2A deletion was shown to be associated with increased sensitivity. Because NF2 and CDKN2A are indeed the genes most frequently mutated in MPM, blocking PI3K/mTOR signaling might be a valid approach to circumvent the difficulty of applying targeted therapy in the absence of an identified oncogene. The rationale for targeting the PI3K/mTOR pathway is also supported by the association of increased activity with a worse clinical outcome.^13, 14^

NVP-BEZ235^(ref15)^ and GDC-0980^(ref16)^ are small-molecule inhibitors of class I PI3K and mTOR (mTORC1 and mTORC2). GDC-0980 has been tested in phase I studies where the phase I extension cohort showed two objective responses among 26 patients with mesothelioma.^17^ Despite these encouraging results, this drug will not be explored further because of side effects observed in another clinical trial.^18^ This, however, should not deter us for trying to find means to improve the antitumor effect of this class of agents. We have previously shown that PI3K/mTOR signaling inhibition sensitizes mesothelioma cells to drugs that are effluxed via ABCG2 transporter by inhibiting the function of ABCG2.^19^ In this study, we aimed at identifying the underlying mechanisms responsible for sensitivity *versus* resistance towards PI3K/mTOR inhibition in a large panel of mesothelioma cell lines. We observed that PI3K/mTOR inhibition increases autophagic rate, which constitutes an efficient mechanism of resistance by inducing growth arrest and survival. However, blocking autophagy, which *per se* affects cell growth, is synthetically lethal when combined with PI3K/mTOR inhibitors by a mechanism involving receptor-interacting protein kinase 1 (RIP1)-dependent cell death.

## Results

### Drug sensitivity screening of mesothelioma cell lines

**Figure 1:**
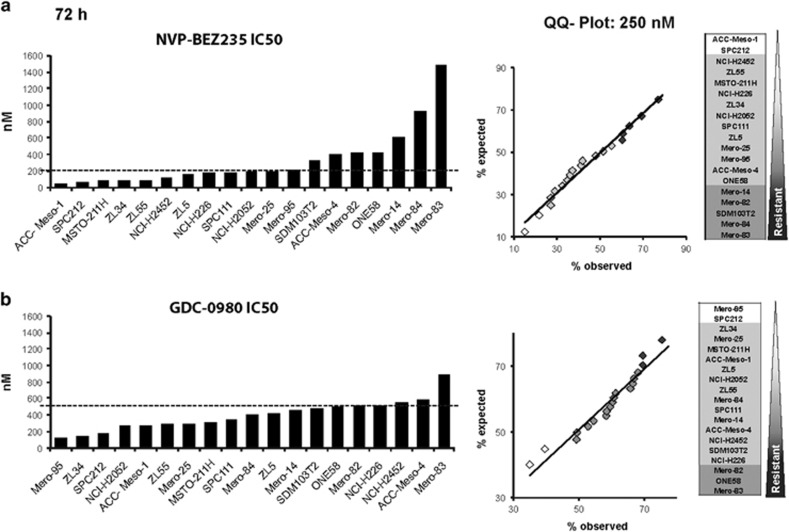
Identification of mesothelioma cell lines sensitive *versus* resistant to PI3K/mTOR inhibition. IC50 of 19 MPM cell lines: ACC-Meso-1, SPC212, MSTO-211H, ZL34, ZL55, NCI-H2452, ZL5, NCI-H226, SPC111, NCI-H2052, Mero-25, Mero-95, SDM103T2, ACC-Meso-4, Mero-82, ONE58, Mero-14, Mero-84 and Mero-83 and Q-Q Plot normality test at 250 nM after 72 h treated with (**a**) NVP-BEZ235 and (**b**) GDC-0980

In this study, we aimed at identifying mechanisms accounting for sensitivity *versus* resistance towards dual PI3K/mTOR inhibitors in a large panel of mesothelioma cell lines. In order to address this question, we performed a cytotoxicity screen in 19 commercially available mesothelioma cell lines. Cells were treated with increasing doses of either NVP-BEZ235 or GDC-0980, and viability and growth inhibition were assayed by measuring mitochondrial activity at 72 h using an MTT assay. The IC50 distribution determined for NVP-BEZ235 showed a difference of about 26-fold between the most sensitive and the most resistant cell lines, whereas GDC-0980 IC50 distribution was more homogenous and showed a maximal difference of 8-fold (Figure 1).

In order to determine whether the results obtained were suitable to select resistant and sensitive cell lines, we tested whether the percentages of growth and viability for a given concentration of either GDC-0980 or NVP-BEZ235 were normally distributed using the Quantile-Quantile Plot (Q-Q Plot) normality test (Figure 1, Supplementary Figure S1). Both NVP-BEZ235 and GDC-0980 cell growth inhibition and viability were normally distributed. The results of viability for NVP-BEZ235 and GDC-0980 were centered at 45.7 and 43.65%, respectively, of untreated control providing maximal sensitivity to detect both resistant and sensitive lines. Thresholds of sensitive and resistant cell lines were set using ±1 S.D. from the mean. The distribution of the cell lines based on the IC50 was consistent to the one observed in the Q-Q Plot.

The published IC50 of NVP-BEZ235 observed in cell lines ranges from 10 to 100 nM^20, 21^ and for GDC-0980 varies between 200 and 500 nM.^22^ The IC50 of 26% of the MPM cell lines treated with NVP-BEZ235 was <100 nM and the IC50 of 68% of MPM cell lines cells treated with GDC-0980 was <500 nM. Based on this observation, the concentration of the drug used for functional assays for NVP-BEZ235 was set to 200 nM, which includes the IC50 of 58% of the cell lines. For GDC-0980, 500 nM was chosen.

### No differences between sensitive and resistant cell lines in PI3K/mTORC1/2 signaling

**Figure 2:**
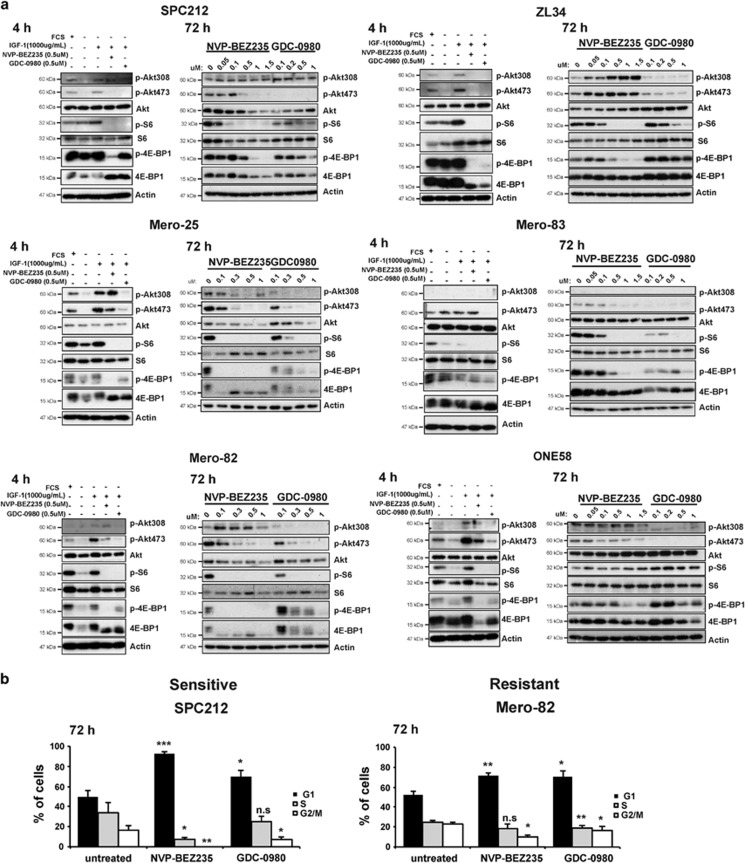
Decrease of PI3K/mTOR signaling is accompanied by G1 arrest, which is more pronounced in sensitive cell lines. (**a**) Protein lysates of sensitive cell lines SPC212, ZL34 and Mero-25 and resistant cell lines Mero-83, Mero-82 and ONE58 were analyzed by western blotting against PI3K/mTOR activity markers: phospho-AKT (Thr308), phospho-AKT (Ser473), AKT, phospho-S6, S6, phospho-4E-BP1, 4E-BP1, and Actin. Each cell line was serum-starved for 16 h and treated as indicated for 4 h with 1000 *μ*g/ml IGF-1, 0.5 *μ*M NVP-BEZ235, 0.5 *μ*M GDC-0980 or treated for 72 h with increasing concentrations of NVP-BEZ235 or GDC-0980 as indicated. (**b**) Cell cycle profile of sensitive cell line SPC212 and resistant line Mero-82 treated with 0.2 *μ*M NVP-BEZ235 or 0.5 *μ*M GDC-0980 for 72 h. Data are presented as means±S.D. from ≥3 independent experiments. Significance was determined by Student's *t*-test (***P<0.005, **P<0.01 and *P<0.05; NS, not significant)

To perform functional assays, six representative cell lines overlapping in sensitivity and resistance to both drugs were chosen, including the sensitive SPC212, ZL34 and Mero-25 and the resistant cell lines Mero-83, Mero-82 and ONE58. We further examined whether PI3K/mTORC1/2 activity markers' phosphorylation was differentially inhibited in sensitive *versus* resistant cell lines and found no substantial differences at the level of mTORC1 signaling (Figure 2a). Both inhibitors similarly reduced the phosphorylation of mTORC1 activity markers 4E-BP1 and S6 already after 4 h of treatment, and this was maintained after 72 h in a dose-dependent manner. S6 phosphorylation did not correlate with 4E-BP1 phosphorylation, and this may be representative on the addiction of cancer cells to aberrant eIF4F heterotrimer-mediated translation,^23^ the assembly of which is inhibited by dephosphorylated 4E-BP1.

GDC-0980 phosphorylation inhibition of mTORC2 substrate AKT (Ser473) was very efficient at 4 h and maintained after 72 h in most of the lines analyzed, whereas NVP-BEZ235 inhibitory effect decreased after 72 h. Similarly, inhibition of the PI3K p100 activity marker phosphorylation AKT (Thr308) was efficient with both drug at 4 h but only the GDC-0980 inhibitory effect was maintained after 72 h for most of the lines analyzed. Similar results with NVP-BEZ235 were observed by Serra *et al.*^21^ in PI3K/mTORC2 inhibition in breast cancer cells where only high concentrations of 500 nM achieved efficient inhibition of PI3K p100. Taken together, although there was no correlation between markers of PI3K/mTORC1/2 activity and the cell growth in sensitive and resistant cell lines for both dual PI3K/mTORC1/2 inhibitors, GDC-0980 was more efficient in blocking PI3K/mTORC2 compared with NVP-BEZ235.

We further investigated the sensitive line SPC212 and the resistant cell line Mero-82 based on their ability to grow in 3D cultures. Cell growth inhibition was confirmed by analyzing the cell cycle profile for each cell line after 72 h of treatment. G1 accumulation with 200 nM NVP-BEZ235 and 500 nM GDC-0980 was significantly more pronounced in the sensitive lines than the resistant cell lines; however, no cell death was observed (Figure 2b, Supplementary Figure S2).

PTEN, a negative regulator of the PI3K pathway, was differentially expressed in all the MPM cell lines tested (Supplementary Figure S3). The sensitive cell lines Mero-25 and ACC-Meso-1 showed no expression of PTEN, indicating that overactivation of PI3K pathway predisposes to sensitivity to the inhibitors.

NF2 loss was shown to sensitize to both PI3K and mTOR inhibitors.^6, 11^ Only four MPM lines expressed NF2 protein (NCI-H2452, ACC-Meso-4, MSTO-211H and Mero-25), consistent with previous studies.^6, 24, 25^ Of the four lines, three (NCI-H2452, ACC-Meso-4, MSTO-211H) have documented wild-type NF2 according to CCLE database and published data.^24, 26^ Because only few lines express proven wild-type NF2, it is difficult to draw any conclusion about any correlation between NF2 status and sensitivity and resistance to dual PI3K/mTOR inhibitors in our experimental settings.

### MPM cell lines have high levels of basal autophagy

**Figure 3:**
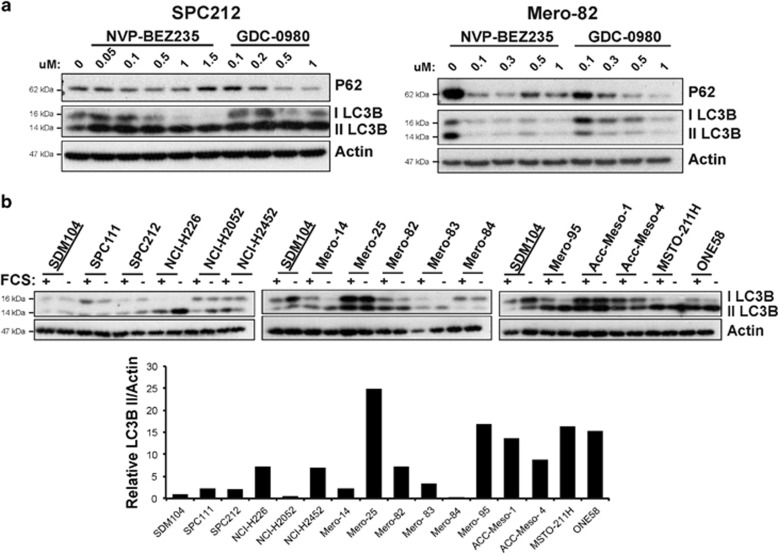
Mesothelioma cells exhibit a high level of autophagy, which is increased upon inhibition of PI3K/mTOR signaling. (**a**) Anti-P62 and -LC3BI/II and -Actin western blots of protein lysates of sensitive cell line SPC212 and resistant cell line Mero-82 treated for 72 h with increasing concentrations of NVP-BEZ235 or GDC-0980 as indicated. (**b**) Anti-LC3BI/II and -Actin western blots of mesothelial cell line SDM104 and MPM cell lines SPC111, SPC212, NCI-H226, NCI-H2052, NCI-H2452, Mero-14, Mero-25, Mero-82, Mero-83, Mero-84, Mero-95, ACC-Meso-1, ACC-Meso-4, MSTO-211H and ONE58 left with serum or serum-starved for 16 h. In the lower panel, protein level quantification of LC3BII normalized against Actin is represented for the cell lines described above with standard culture conditions

Autophagy is a survival mechanism by which cytosolic material is sequestered in a double-layered membrane, autophagosome, delivered to the lysosome for degradation and recycled to fuel cellular growth in periods of starvation or distress.^27^ Cancer can take advantage of this mechanism to maintain cell viability, leading to tumor dormancy, progression and therapeutic resistance.^28^ Consistently with the observations done by others that NVP-BEZ235 induces autophagy in glioma cells^29, 30, 31^ and GDC-0980 in pancreatic cancer,^32^ we also observed sustained autophagy induction in SPC212 and Mero-82 cells after 72 h of treatment, evidenced by the processing of LC3-I to the autophagosome localized LC3-II form and P62 degradation (Figure 3a). Therefore, we hypothesized that autophagy could act as resistance mechanism in our mesothelioma model. We found that almost all the mesothelioma cells analyzed had high levels of basal autophagy as indicated by LC3-II form (Figure 3b) as compared with the non-transformed mesothelial cell line SDM104. Overnight serum starvation did not considerably increase the level of basal autophagy in most of the cell lines analyzed, in contrast to other metabolism adjustments observed after external growth factor deprivation.^33^ No correlations between levels of LC3-II and changes of PI3K/mTOR signaling regulators such as PTEN, NF2 as well as mTORC1 activity (documented by S6 phosphorylation) between resistant and sensitive lines were observed in serum-deprivation conditions (Supplementary Figure S3).

### SPC212 and Mero-82 cells have a high autophagic flux and autophagy blockade sensitizes them to PI3K/mTOR inhibitors inducing cell death in a RIP1-dependent manner

GDC-0980 and NVP-BEZ235 alone or in combination with other chemotherapeutics or radiotherapy have been shown to induce intrinsic apoptosis in a variety of solid and liquid tumors.^20, 22, 29, 34, 35, 36^ Moreover, in cancers, such as renal cell carcinoma or pancreatic cancer, the combination of autophagy inhibitor chloroquine (CQ) with single mTOR or dual PI3K/mTOR inhibitors sensitizes the cells and induces caspase-dependent and -independent cell death.^32, 37, 38, 39^ CQ is a lysosomotropic drug that raises intralysosomal pH^40^ and impairs autophagic protein degradation.

**Figure 4:**
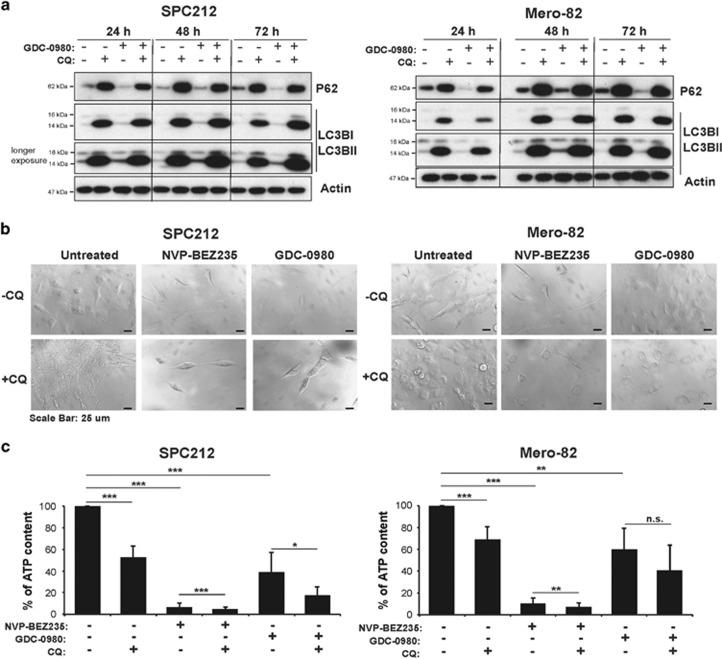
Inhibition of autophagy with CQ sensitizes mesothelioma cell lines to PI3K/mTOR inhibitors. (**a**) Anti-P62, L-C3BI/II and Actin western blots of protein lysates of sensitive cell line SPC212 and resistant cell line Mero-82 treated with 20 *μ*M CQ, 0.5 *μ*M GDC-0980 or in combination for 24, 48 and 72 h. (**b**) Light micrographs of SPC212 and Mero-82 treated with 0.2 *μ*M NVP-BEZ235, 0.5 *μ*M GDC-0980 alone or in combination with 20 *μ*M CQ for 96 h. Scale bar represents 25 *μ*m. (**c**) Quantification of the percentage of ATP content of SPC212 cells and Mero-82 cell treated with 0.2 *μ*M NVP-BEZ235, 0.5 *μ*M GDC-0980 alone or in combination with 20 *μ*M CQ for 96 h. Data are presented as means±S.D. from ≥3 independent experiments. Significance was determined by analysis of variance test (***P<0.005, **P<0.01 and *P<0.05; NS, not significant)

In order to test whether NVP-BEZ235 and GDC-0980 promote survival via autophagy, sensitive and resistant lines were treated with the inhibitors alone or in combination with CQ. CQ alone and in combination with GDC-0980 efficiently blocked autophagy after 24 h of treatment, and this effect was maintained after 72 h. GDC-0980 alone increased the autophagic flux in SPC212 and Mero-82 cells at 72 h (Figure 4a). Strong cytoplasm vacuolization was induced with CQ treatment alone or in combination with the inhibitors in both cell lines, supporting the results from the immunoblotting (Figure 4b). Although a strong morphological impact of CQ combined with dual PI3K/mTOR inhibitors was observed at earlier time points, cell death was detected only after 96 h in the cell lines tested (Figure 4c, Supplementary Figure S4). We observed a significant ATP drop in both cell lines treated with CQ alone, and this was less prominent in the resistant line Mero-82 after 96 h of treatment. The combination showed a significant additive effect in the reducing ATP levels in SPC212 cells and a minor effect in Mero-82 (Figure 4c), which was confirmed by colony-formation assay in Mero-82 cells (Supplementary Figure S5).

**Figure 5:**
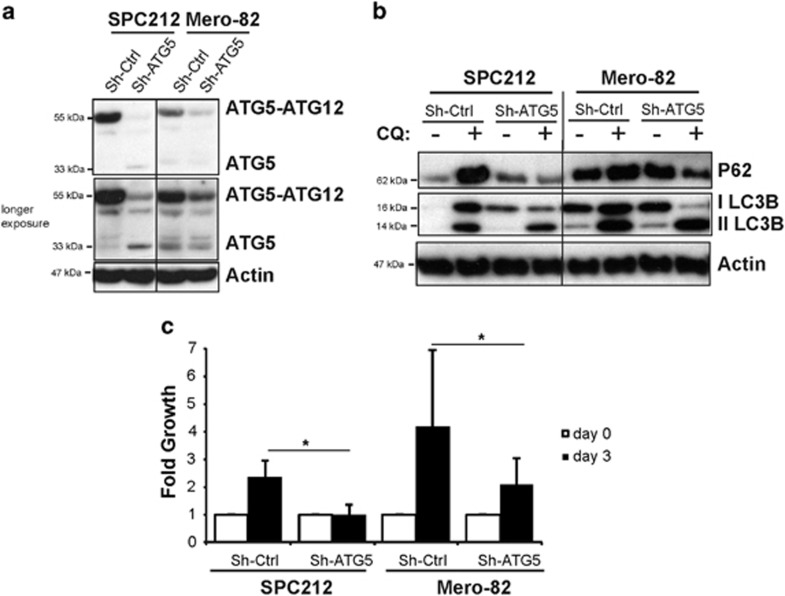
Autophagy is necessary for mesothelioma cell growth. SPC212 and Mero-82 cell lines were stable transduced with virus particles carrying Mir30- Sh-Atg5 or Mir30-Sh-Ctrl. (**a**) Atg5 gene silencing was confirmed by anti-ATG5 western blotting. (**b**) Anti-P62, -LC3BI/II and -Actin of SPC212 and Mero-82 Sh-Atg5 and Sh-Ctrl untreated or treated with 20 *μ*M CQ for 4 h. (**c**) Fold cell growth of SPC212 and Mero-82 Sh-Atg5 and Sh-Ctrl for 3 days. Data are presented as means±S.D. from three independent experiments. Significance was determined by Mann–Whitney *U*-test (*P<0.05)

In order to confirm the role of autophagy in sensitization to PI3K/mTOR inhibition, we silenced the Atg5 gene (Figure 5a), which is important for the autophagosome elongation. Silencing of Atg5 resulted in abolition of P62 accumulation after CQ treatment (Figure 5b), thus demonstrating efficient blockade of autophagy. Similar results were observed with a second short hairpin system (data not shown). Surprisingly, silencing Atg5 gene was associated with significantly impaired cell growth in SPC212 sensitive line and Mero-82 resistant line (Figure 5c), indicating the dependency of the MPM on autophagy. Because of this phenotype, we could not test the effect of PI3K/mTOR inhibition.

**Figure 6:**
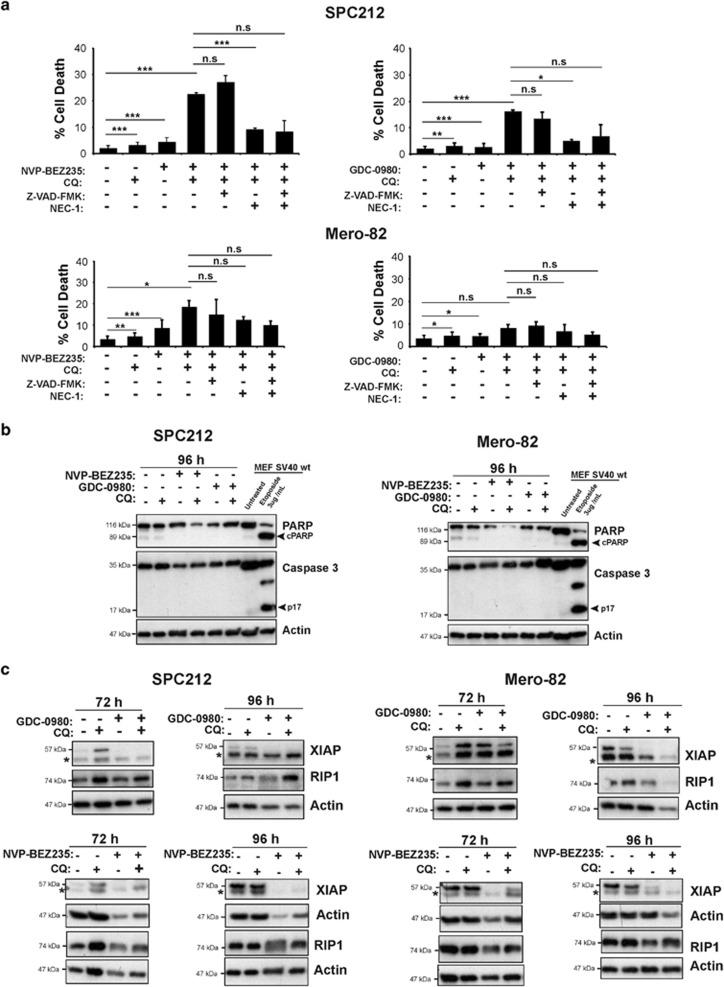
Inhibition of autophagy with CQ combined with inhibition of PI3K/mTOR signaling induces caspase-independent cell death. (**a**) SPC212 and Mero-82 cell lines were treated as indicated with 0.2 *μ*M NVP-BEZ235, 0.5 *μ*M GDC-0980, 20 *μ*M CQ, 20 *μ*M ZVAD-FMK or 50 *μ*M Nec-1 for 96 h. Cell death was assessed by GFP-Annexin V/PI staining and flow cytometry. Data are presented as means±S.D. from three independent experiments. Significance was determined by analysis of variance test (***P<0.005, **P<0.01 and *P<0.05; NS, not significant). (**b**) Anti- PARP, -Caspase 3 and -Actin western blots of SPC212 and Mero-82 treated as indicated with 0.2 *μ*M NVP-BEZ235, 0.5 *μ*M GDC-0980 and 20 *μ*M CQ. SV40 immortalized wt MEFs treated for 15 h with 3 *μ*g/ml Etoposide were used as control for apoptosis. (**c**) Anti-XIAP (asterisk (*) represents unspecific band), -RIP1 and -Actin western blots of protein lysates of SPC212 and Mero-82 treated as indicated with 0.2 *μ*M NVP-BEZ235, 0.5 *μ*M GDC-0980 and 20 *μ*M CQ for 72 and 96 h

To determine the type of cell death induced by the combination of CQ and the dual PI3K/mTOR inhibitors, cells were examined for apoptosis by assessing the Annexin V/PI viability assay (Figure 6, Supplementary Figure S6), effector caspase 3 processing and cleavage of the caspase 3 substrate PARP. We observed cell death induction in both SPC212 and Mero-82 cells with both inhibitors in the Annexin V/PI assay; however, significant higher cell death induction was only achieved when CQ was added. This additive effect was bigger in the sensitive line SPC212 than in the resistant line Mero-82. Addition of the broad spectrum pan-caspase inhibitor Z-VAD-FMK failed to rescue cell death (Figure 6a). No active caspase-3, PARP cleavage or cytochrome *c* release was detected upon combined treatment (Figure 6b, Supplementary Figure S7), and we also did not observed an increase of the BH3 only protein BIM (data not shown). Altogether, these observations support that intrinsic apoptosis was not involved. We then checked whether the death observed may occur via RIP1-dependent mechanism.^41^ SPC212 and Mero-82 cells were treated with CQ and NVP-BEZ235 or GDC-0980 in the absence and presence of the specific RIP1 inhibitor Necrostatin-1 (Nec-1).^42^ Nec-1 alone was able to significantly block the cell death induced by CQ and the dual PI3K/mTOR inhibitors in SPC212. In Mero-82 cells, Nec-1 did not block cell death. Furthermore, the dual PI3K/mTOR inhibitors induced RIP1 degradation (Figure 6c), which was then rescued by the addition of CQ in both cell lines. This effect was more obvious after 96 h, when the cells started to die. Interestingly, prosurvival protein X-linked inhibitor of apoptosis protein (XIAP) was efficiently downregulated as a result of AKT inhibition only in the sensitive line SPC212 but not in the resistant line Mero-82.

A number of crosstalk, feedback and feed-forward loops link the PI3K/AKT/mTOR and Ras/MEK/ERK signaling pathways, which provide insights into the compensatory responses observed with targeting either pathway alone.^43^ We observed that GDC-0980 induced activation of ERK in resistant Mero-82 cells but not in sensitive SPC212 cells (Supplementary Figure S8a). ERK activation was also strongly induced by CQ in resistant Mero-82 cells. To test whether inhibiting the MEK/ERK pathway would enhance the effect of GDC-0980 and CQ, we tested U0126, a MEK1/2 inhibitor. U0126 efficiently inhibited ERK induction by CQ and GDC-0980 (Supplementary Figure S8b). However, combination of U0126, GDC-0980 and CQ did not induce cell death (Supplementary Figure S8c).

### Dual PI3K/mTOR inhibitors in combination with CQ induce cell death in 3D MPM

Spheroids have been observed in the pleural fluid of human MPM and linked to increased malignancy.^44^ MPM spheroids have been used to investigate new therapeutic options^45^ and have shown that this model better represent biological complexity existing in patients' tumor.^46, 47^ Therefore we aimed at verifying in this model the efficiency of blocking PI3K/mTOR combined with inhibition of autophagy.

In this study, we used 300-*μ*m diameter densely packed spheroids formed in 4 days according to a standardized protocol.^48^ In these conditions, about one-third of the cells are in quiescent state,^44^ thereby corresponding better to proliferation status of tumoral cells^49^ compared with 2D cell culture.

**Figure 7:**
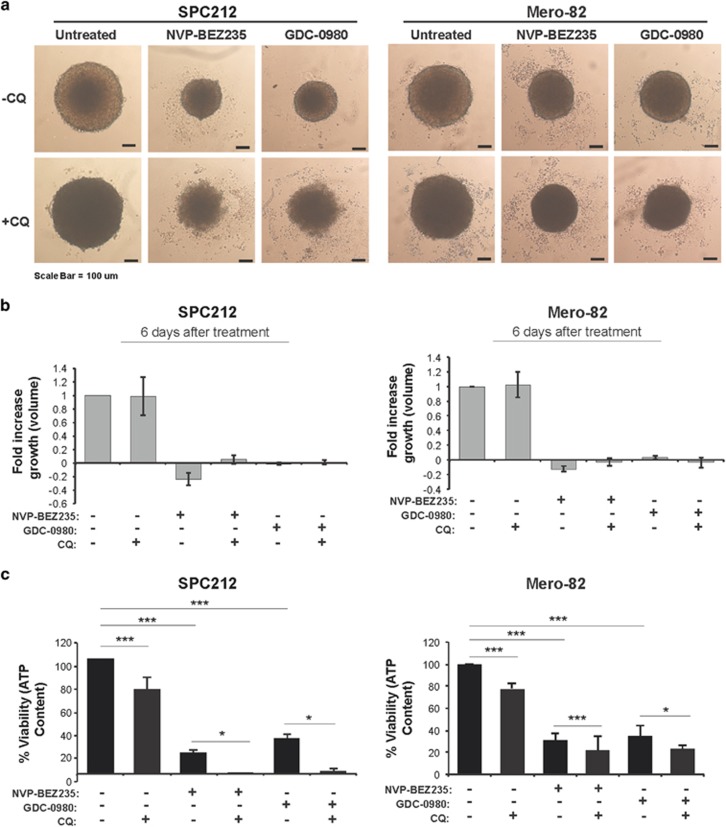
Inhibition of autophagy with CQ combined with inhibition of PI3K/mTOR signaling induces spheroid cell death. (**a**) Representative light micrographs of SPC212 and Mero-82 spheroids treated as indicated with 1 *μ*M NVP-BEZ235, 1 *μ*M GDC-0980 and 20 *μ*M CQ for 6 days. (**b**) Fold increase volume (growth) of spheroids shown in panel (**a**). (**c**) Viability is presented as the percentage of ATP content in SPC212 and Mero-82 spheroids treated as described in panel (**a**). Data are presented as means±S.D. from ≥3 independent experiments. Significance was determined by analysis of variance test (***P<0.005 and *P<0.05; NS, not significant)

Mero-82 and SPC212 spheroids were treated either with CQ alone or in combination with NVP-BEZ235 or GDC-0980. Although treatment with CQ alone did not have an impact on spheroid growth, there was a significant drop in ATP content of around 20% for both the cell lines (Figures 7a–c). In addition, the spheroids were refractive and appeared dark in light microscopy. Treatment of the spheroids with the PI3K/mTOR inhibitors alone induced significant cell growth inhibition and ATP drop in both the cells lines (Figures 7b and c). Combination of CQ with PI3K/mTOR inhibitors did not have an additional impact on cell growth *per se*; however, strong cytoplasm vacuolization and swelling of the single cells (Figure 7a) was observed. Moreover, there was a significant additive effect on ATP reduction for both the cell lines. Interestingly, in the SPC212 spheroids ATP content was almost depleted and they appeared disintegrated and individual cells were recognizable while Mero-82 the spheroid shape was still intact at the time point of analysis.

Inhibition of autophagy did not sensitize normal SDM104 cells to PI3K/mTOR inhibition (Supplementary Figures S9a–c).

In order to test whether ERK pathway is involved in Mero-82 resistance in 3D, we added U0126 (Supplementary Figure S10). Combination of U0126 and GDC-0980 had a slight additive effect on viability, and there was no additional significant effect when CQ was added. Disintegration of the spheroids was not observed after 6 days of treatment. These results indicate that ERK is not the major pathway involved in cell death resistance.

## Discussion

In this study, we demonstrate that shortcut escape into autophagy is one way to improve therapeutic efficiency of PI3K/mTOR inhibition in MPM.

Several mechanisms have been described to underlie resistance to PI3K/mTOR inhibition. For example, breast cancer cell lines resistant to NVP-BEZ235 have increased JAK2/STAT5 activation resulting from IL-8 secretion.^50^ Along these lines, combined treatment with GDC-0980 together with a c-Met inhibitor efficiently inhibited mesothelioma tumor growth *in vivo*.^51^ Dual PI3K/mTOR inhibitors were described^52^ to induce ERK activation by inhibiting mTORC2 in pancreatic cancer. Combination of PI3K/mTOR and MEK/ERK inhibitors enhanced the drug response. We observed a similar effect in the resistant cell line Mero-82; however, the combination of PI3K/mTOR and MEK/ERK inhibitors, in the presence or absence of autophagy inhibition, did not result in cell death, indicating that an additional mechanism might be involved. Another known resistance mechanisms to PI3K/mTOR inhibition include cap-independent translation and FOXO-mediated mechanism.^53^ NVP-BEZ235 was shown to induce upregulation and/or activation of several prosurvival proteins such as RTK, cytosolic kinases, antiapoptotic proteins and transcription factors mostly by cap-independent translation.^54^ mRNAs translated under these conditions have a highly structured 50-untranslated region, which often harbors an Internal Ribosome Entry Site sequence and several upstream AUGs. One of the protein that is translated in this way under conditions of reduced global protein synthesis is XIAP.^55^ XIAP is a key intrinsic regulator of apoptosis, primarily by virtue of its ability to bind to and inhibit both initiator and effector caspases.^56^ Although the cellular levels of XIAP are regulated by several independent mechanisms, the predominant regulation appears to be the control of XIAP mRNA translation. Levels of XIAP were upregulated by GDC-0980 in Mero-82 resistant but not in SPC212 sensitive cells. Interestingly, XIAP levels were also upregulated by CQ, but this might be related to other mechanisms, including autophagy inhibition-driven increase in the levels of proteasome substrates.^57^

FOXO-dependent mechanisms may underlie the different response to the two PI3K/mTOR inhibitors used in this study. Indeed, mTORC2 was more efficiently inhibited by GDC-0980 compared with NVP-BEZ235, resulting in inhibition of AKT activation. Atg genes are positively controlled by FoxO3a,^58^ and the latter is negatively regulated by active AKT. Therefore GDC-0980 by inducing a better blockage of AKT activation may result in long-term, FOXO3a-dependent commitment to autophagy, and indeed we observed decreased phosphorylation of FOXO3a after GDC-0980 treatment (data not shown). Our observations are consistent with increased efficiency observed by inhibition of autophagy together with dual PI3K/mTOR inhibitors in pancreatic cancer^59^ and peripheral nerve sheath tumors.^60^

The cell death observed in our experimental settings is most likely necroptosis, which is one of the several forms of regulated cell death involving RIP1, RIP3 and MLKL.^41^ The mechanism activating necroptosis include dropping ATP levels, which at some point abolish the activity of all ATP-dependent enzymes (including various transporters that maintain ionic balance at the plasma membrane) and a compromised redox balance (which inactivates various enzymes and causes oxidative molecular damage to organelles and membranes) as central players in the execution of regulated cell death.^41^ We documented a drastic decrease of ATP levels which supports the theory that cells died due to necroptosis. The decrease of ATP level was more severe in the sensitive SPC212 cells compared with the resistant Mero-82 cells. Interestingly, cell death induced by combined treatment was efficiently blocked by RIP1 inhibitor Nec-1 only in the sensitive cell line SPC212. This indicates that the actual induction of cell death in the resistant Mero-82 line happens in a RIP1-independent context, most probably involving only RIP3 and MLKL.^41^ The likelihood of this scenario is suggested by recent data showing that XIAP inhibits TNF- and RIP3-dependent cell death^61^ and by our observation that XIAP was not efficiently downregulated upon combined drug treatment in Mero-82 compared with SPC212 cells

The results observed were consistent in the 2D and 3D models. This is important as baseline phosphorylation of PI3K/AKT/mTOR pathway members have been described to be reduced in 3D spheroids compared with 2D.^47^ Because 3D/spheroids represent a closer model to tumor biological complexity, these results suggest that this combined therapeutic option should be explored in clinical settings.

In addition, we observed that inhibition of autophagy *per se* had an effect on mesothelioma cell growth. Autophagy promotes the survival of cells resistant to apoptosis when they are deprived of extracellular nutrients or growth factors. Treatment of such cells dependent on autophagy for survival with CQ results in ATP-depletion-dependent cell death.^62^ Inhibition of cancer cells' intrinsic autophagy by silencing Atg5 decreases B16-F10 tumor growth.^63^ Autophagy has a context-dependent role in cancer.^64^ It is upregulated and required for the survival of tumor cells in hypoxic tumor regions.^64^ Knocking out essential autophagy genes in genetically engineered mouse models for cancer have demonstrated a pro-tumorigenic role for autophagy.^65^ The mechanisms behind 'autophagy addiction' observed in MPM cells are beyond the scope of present study but will be further explored.

In conclusion, we demonstrated the PI3K/mTOR inhibitors increase autophagy levels in MPM and combination together with CQ should be further explored as inhibition of autophagy blocks this resistance mechanism.

## Materials and Methods

### Reagents

Dulbecco's Modified Eagle's Medium/Nutrient Mixture F-12 Ham (DMEM–F12), propidium iodide and Penicillin/Streptomycin 100 × stock solution were purchased from Sigma-Aldrich Chemie GmbH (Buchs, Switzerland). Trypsin solution 0.25% was purchased from GIBCO (Life Technologies Europe, Zug, Switzerland). Fetal calf serum (FCS, CVFSVF00-01) was purchased from Eurobio (Ulis (Les), France). Puromycin was purchased from AppliChem (Darmstadt, Germany). Z-Val-Ala-DL-Asp-fluoromethylketone (Z-VAD-FMK) was purchased from BACHEM (Bubendorf, CH, Switzerland). Nec-1 was purchased Enzo LifeSciences AG (Lausen, Switzerland). NVP-BEZ235 was obtained from Novartis (Basel, Switzerland) and GDC-0980 was obtained from Genentech (Roche, Basel, Switzerland). U0126 was purchased from Cell Signaling Technology (Allschwil, Switzerland). Nivaquine (chloroquine sulfate) was purchased from Sanofi Aventis (Paris, France). Recombinant His_6_-GFP-Annexin V was kindly provided by T Kaufmann (Bern, CH, Switzerland).

### Cell culture

The following mesothelioma cell lines have been used: ZL55, ZL5, ZL34, SDM103T2, SPC111 and SPC212 from our laboratory; NCI-H226, NCI-H2052, NCI-H2452, MSTO-211H^66^ were obtained from ATCC (Wesel, Germany); ACC-Meso-1 and ACC-Meso-4^24^ were obtained from Riken BRC (Ibaraki, Japan); and Mero-25, Mero-82, Mero-83, Mero-84, Mero-95^(ref 67)^ and ONE58^(ref 68)^ was obtained from the European Collection of Cell Cultures (Salisbury, UK). The non-transformed mesothelial cell line SDM104^(ref 69)^ was established in our laboratory. Cells established in our laboratory were maintained as described by Thurneysen *et al.*^70^ The rest of the cell lines were cultured in DMEM–F12 supplemented with 15% FCS and 1% Penicillin/Streptomycin solution. SV40 immortalized wild-type MEF cells and Hek 293T cells were cultured in DMEM high glucose medium supplemented with 10% FCS and 1% Penicillin/Streptomycin. All cells were cultured at 37 °C in a humidified 5% CO_2_ atmosphere.

### Gel electrophoresis and immunoblotting

Total protein extracts were prepared by lysing the cells with hot H8 buffer containing 10 mM Tris-Cl pH 7.4, 2 mM EDTA, 2 mM EGTA and 1% SDS and boiled for 5 min. Protein concentration was determined using a DC Protein Assay (Bio-Rad, Hercules, CA, USA), and proteins were prepared by adding 6 × reducing Lämmli buffer (600 mM DTT) and boiling the samples for 5 min. A total of 10 *μ*g protein per extract was separated on denaturing 4–20% gradient SDS-PAGE gels. Proteins were transferred onto PVDF transfer membranes (0.45 *μ*m, Perkin Elmer, Schwerzenbach, Switzerland). For western blotting, membranes were probed with the following primary antibodies: rabbit anti-AKT (no. 9272), rabbit anti-Phospho-AKT (Thr308) (no. 9275), rabbit anti-Phospho-AKT (Ser473) (193H12, no. 9275), mouse anti-S6 (54D2, no. 2317), rabbit anti-Phospho-S6 (Ser235/236) (D57.2.2E, no. 4858), rabbit anti-4E-BP1 (no. 9452), rabbit-Phospho-4E-BP1 (Thr37/46) (236B4, no. 2855), rabbit anti-p44/42 MAP Kinase (no. 9102), rabbit anti-Phospho-p44/42 MAP Kinase (Thr202/Tyr 204) (no. 9101), rabbit anti-PTEN (138G6, no. 9559), rabbit anti-LC3B (no. 2775), rabbit anti-PARP (no. 9542), rabbit anti-caspase-3 (no. 9662) from Cell Signaling Technology; rabbit anti-NF2 (C18, no. sc-332) from Santa Cruz Biotechnologies (Dallas, TX, USA); guinea pig anti-p62 (GP62-C) from Progen (Heidelberg, Germany); mouse anti-RIP (no. 610458) and mouse anti-XIAP (no. 610763) from Transduction Laboratories (Allschwil, Switzerland); mouse anti-ATG5 (7C6) from Nanotools (München, Switzerland); rabbit anti-Phospho-NF2 (Ser518) (PAI-14252) from Pierce (Rockford, IL, USA) and mouse anti-Actin (no. 69100) from MP Biomedicals (Santa Ana, CA, USA). Membranes were then incubated with the secondary antibody goat anti-mouse IgG-HRP (A-5420) from Ancell (Bayport, MN, USA), goat anti-guinea pig IgG-HRP (sc-2438) from Santa Cruz and goat anti-rabbit IgG-HRP (no. 7074) from Cell Signaling. The signals were detected by enhanced chemiluminescence (ECL Western Blotting Reagents, GE Healthcare, Glattbrugg, Switzerland) and detected on photosensitive film (Super RX Fuji x-Ray Film, Fujifilm, Düsseldorf, Germany).

### Quantification of cell death by flow cytometry

Floating cells and attached cells were collected and washed with Annexin V staining buffer (150 mM NaCl, 4 mM KCl, 2.5 mM CaCl2, 1 mM MgSO4, 15 mM HEPES pH 7.2, 2% FCS and 10 mM NaN3) and incubated with GFP-Annexin V diluted in staining buffer for at least 30 min on ice in the dark. Cells were then washed in Annexin V staining buffer and resuspended in 500 *μ*l staining buffer. Propidium iodide was added to a final concentration of 2 *μ*g/ml, and cells were examined using on an Attune flow cytometer (Applied Biosystems, Zug, Switzerland) and analyzed with the Attune cytometric software v1.2.5 (Applied Biosystems). GFP-Annexin V- and propidium iodide-positive cells were considered as dead cells.

### MTT assay and IC50 calculation

Cells were plated in sextuplicates at a density of 3000 cells/well in 96-well plates and allowed to adhere overnight followed by serum starvation for 16 h. The cells were then exposed to 0, 0.001, 0.01, 0.05, 0.1, 0.25, 0.5, 1 and 5 *μ*M of GDC-0980 or NVP-BEZ235 for 1 h in serum-free condition, and medium containing serum and the inhibitors were added to the cells and incubated for 72 h. Mitochondrial activity as a readout for cellular viability was assessed by MTT assay. In all, 10 *μ*l of a 10 mg/ml MTT stock solution (3-amino-9-ethyl-carbazole, Sigma Aldrich, Buchs, Switzerland) was added per well containing cells in 100 *μ*l DMEM–F12 without Phenol Red. Plates were incubated for 90 min at 37 °C and cells lysed with 100 *μ*l MTT lysis buffer (10% SDS, 45% dimethyl formamide, adjusted to pH 4.7 by 80% acetic acid/1 M HCl) for 2 h. Absorbance was measured at 570 nm using SpectraMax microplate reader (Molecular Devices, Sunnyvale, CA, USA). IC50 was calculated using the GraphPad Prism Software (GraphPad Inc., San Diego, CA, USA). Cell growth inhibition was assessed using the formula 100 × (*t*−*t*0)/(*c*−*t*0)=50, where ‘*t*' is the OD of 72-h exposure time to the test drug, ‘*t*0' the OD at time 0 and ‘*c*' the control OD in the absence of drugs.

### Cell cycle analysis

For cell cycle analysis, floating cells and attached cells were collected, washed with D-PBS and fixed with ice cold 70% ethanol on ice for 30 min. Then cells were washed twice with D-PBS and incubated with PI/RNAse solution (50 *μ*g/ml and 100 *μ*g/ml) at RT for 1 h. Cells were analyzed on an Attune flow cytometer (Applied Biosystems) and data were processed using the ModFit LT cell cycle software (Topsham, ME, USA).

### Spheroid formation

To form spheroids, 1000 cells/well for SPC212 and 2000 cells/well for Mero-82 per well were plated on ultra-low attachment 96-well round-bottomed plates (Sigma-Aldrich). Cells were concentrated by gentle centrifugation at 300 × *g* for 5 min and incubated at 37 °C for 4 days in order to reach 250–300 *μ*m before starting the treatment. For each treatment, triplicates were performed. After 6 days, volume of the spheroid was calculated by measuring the spheroid diameter on light micrographs. Spheroid growth fold increase was assessed using the formula (*t*−*t*0)/(*c*−*t*0); ‘*t*'=spheroid volume after 6 days of exposure to test drug, ‘*t*0'=spheroid volume at time 0 and ‘*c*'=control spheroid volume after 6 days. Viability was performed by determining the ATP content of the spheroids using CellTiter-Glo Luminescent Cell Viability Assay (D160963, Promega, Dübendorf, Switzerland), following the manufacturer's instructions. Luminescence was acquired using GloMax 96 Microplate Luminometer (Promega).

### Lentiviral gene transfer and gene silencing

Two types of short hairpins targeting Atg5 and their respective controls were used: Atg5 shRNA was subcloned in pMSCV-Puro-miR30^(ref 71)^ and pLKO.1 ATG5 shRNA (Sigma-Aldrich), which were kindly provided by Dr. HU Simon (Bern, CH, Switzerland). HEK 293T cells were co-transfected with PMD2.VSV-G (envelope coding sequence): psPAX2 (packaging elements): shRNA Atg5 or control (total of 5 *μ*g of DNA) at a ratio of 2:5:3 in 10-cm tissue culture dishes using Lipofectamine 2000 Transfection Reagent (Life Technologies). Lentiviral particles were harvested from the medium 24 and 48 h later, pooled and passed through a 0.2-*μ*m filter and used fresh for infection or stored in aliquots at −80 °C. Target cell lines were transduced in the presence of 8 *μ*g/ml polybrene. Successfully transduced cells were selected with 0.3 *μ*g/ml puromycin for a minimum of 3 weeks.

### Statistical analysis

IC50 was calculated using the GraphPad Prism version 5.0 (GraphPad Inc.). Q-Q Plot was used to identify sensitive and resistant cell lines. Drug response viability of the cell lines >1 S.D. from the mean were considered resistant, and the drug response viability of cell lines <1 S.D. from the mean were considered sensitive. Statistical analyses were performed using StatView version 5.0.1 (SAS Institute, Cary, NC, USA). In order to evaluate statistically significant differences between normally distributed variables paired, Student's *t*-test was used. To assess significance between variables, which were not normally distributed, Mann–Whitney *U*-test was used. To determine significant additive effects between combination treatments and inhibition of cell death with the inhibitors, ANOVA test was performed. Differences were considered significant when *P*<0.05.

## Abbreviations

mTOR: mammalian target of rapamycin
PI3K: phosphatidylinositol 3-kinase
RIPK1: receptor-interacting protein kinase 1
XIAP: X-linked inhibitor of apoptosis protein
nec-1: necrostatin-1
