# Supplementary material for: Inhibition of autophagy sensitizes malignant pleural mesothelioma cells to dual PI3K/mTOR inhibitors
Source: Cell Death Dis. 2015 May 7;6(5):e1757–. doi: 10.1038/cddis.2015.124 (PMC4669703; doi:10.1038/cddis.2015.124)
Supplement: Supplementary Information [file cddis2015124x1.docx]

**Legends to Supplementary Figures**

**Supplementary Figure 1. Identification of mesothelioma cell lines sensitive vs. resistant to PI3K/mTOR inhibition based on cell growth inhibition**

QQ-Plot normality of 19 Mesothelioma cell lines: ACC-Meso-1, SPC212, MSTO-211H, ZL34, ZL55, NCI-H2452, ZL5, NCI-H226, SPC111, NCI-H2052, Mero-25, Mero-95, SDM103T2, ACC-Meso-4, Mero-82, ONE58, Mero-14, Mero-84 and Mero-83 at 250 nM cell growth inhibition after 72 h NVP-BEZ235 and GDC-0980 treatment.

**Supplementary Figure 2. Cell cycle profile of Mero-25, ONE58 and Mero-83 cells**

Cell cycle profile of Mero-25, ONE58 and Mero-83 after 0.2 µM NVP-BEZ235 and 0.5 µM GDC-0980 treatment for 72 h. Data are presented as means 2 independent experiments.

**Supplementary Figure 3. PTEN, NF2 and Phospho-S6 protein levels in MPM cell lines**

Anti-PTEN, -phospho-S6, -S6, -phospho-NF2, -NF2 and -Actin western blots of protein lysates of mesothelial cell lines SDM104 and SDM85; and MPM cell lines SPC111, SPC212, NCI-H226, NCI-H2052, NCI-H2452, Mero-14, Mero-25, Mero-82, Mero-83, Mero-84, Mero-95, ACC-Meso-1, ACC-Meso-4, MSTO-211H and ONE58 left with serum or serum-starved for 16 h.

**Supplementary Figure 4. Treatment with dual PI3K/mTOR inhibitors in combination with CQ induce cell death in MPM cell lines.**

SPC212 and Mero-82 cell lines were treated as indicated with 0.2 µM NVP-BEZ235, 0.5 µM GDC-0980; and 10, 15 and 20 µM CQ for 96 and 120 h. Cell death was assessed by GFP-Annexin V/PI staining and flow cytometry. Data are presented as means from two independent experiments.

**Supplementary Figure 5. Colony formation assay of Mero-82 cells**

Cristal violet staining of colonies formed from 1000 Mero-82 cells. Cells were serum starved for 16 h and treated with 0.2 µM NVP-BEZ235, 0.5 µM GDC-0980 and 20 µM CQ as indicated. Medium without the inhibitors was added after 24 h and cells were incubated for 6 days more.

**Supplementary Figure 6. Treatment with dual PI3K/mTOR inhibitors in combination with CQ induce cell death in MPM cell lines.**

GFP-Annexin V/PI staining of ACC-Meso-4, ACC-Meso-1, Mero-25, SPC212 and Mero-82 cell lines treated as indicated with 0.2 µM NVP-BEZ235, 0.5 µM GDC-0980 and 20 µM CQ for 96 h. Cell death was assessed by GFP-Annexin V/PI staining and flow cytometry.

**Supplementary Figure 7. Treatment with dual PI3K/mTOR inhibitors in combination with CQ does not induce cytochrome c release in SPC212 and Mero-82 cells.**

Fluorescence micrographs of cytochrome c (green) immuno- and DAPI- (blue) staining of SPC212 and Mero-82 cell lines treated as indicated with 0.2 µM NVP-BEZ235, 0.5 µM GDC-0980 and 20 µM CQ for 96 h.

**Supplementary Figure 8. Inhibition of ERK does not decrease resistance of Mero-82 cells to dual PI3K/mTOR inhibitors in combination with CQ .**

(a) ERK is activated in Mero-82 cells upon treatment with dual PI3K/mTOR inhibitors and in combination with CQ. Anti-phospho ERK, -ERK and -Actin western blots of protein lysates of sensitive cell line SPC212 and resistant cell line Mero-82 treated as indicated with 20 µM CQ and 0.5 µM GDC-0980 for 120 h. (b) U0126 inhibits ERK activation induced upon treatment with dual PI3K/mTOR inhibitors and in combination with CQ. Protein lysates of Mero-82 cells treated as indicated with 0.5 µM GDC-0980, 20 µM U0126 and 20 µM CQ for 96h. (c) Mero-82 cell line was treated as indicated with 0.5 µM GDC-0980, 20 µM U0126 and 20 µM CQ 96 h. Cell death was assessed by GFP-Annexin V/PI staining and flow cytometry. Data are presented as means +/- SD from three independent experiments. Significance was determined by Anova test.

**Supplementary Figure 9. PI3K/mTOR in combination with CQ does not induce cell death in SDM104 spheroids**

(a) Protein lysates of non-transformed mesothelial cell line SDM104 were analyzed by western blotting against PI3K/mTOR activity markers: phospho-AKT (Thr308), phospho-AKT (Ser473), AKT, phospho-S6, S6, phospho-4E-BP1, 4E-BP1 and Actin. SDM104 was serum-starved for 16 h and treated as indicated 0.2 µM NVP-BEZ235, 0.5 µM GDC-0980 for 72 h (b) Representative light micrographs of SDM104 spheroids treated as indicated with 1 µM NVP-BEZ235, 1 µM GDC-0980 and 20 µM CQ for 6 days. (c) Viability is presented as percentage of ATP content SPC212 and Mero-82 spheroids treated as described in (b). Data are presented as means +/- SD from ≥3 independent experiments. Significance was determined by Anova test (***p<0.005; n.s.: not significant)..

**Supplementary Figure 10. Inhibition of ERK/mTOR/PI3K and autophagy does not induce cell death in the resistant cell line Mero-82 spheroids**

(a) Representative light micrographs of Mero-82 spheroids treated as indicated with 1 µM GDC-0980, 20 µM U0126 and 20 µM CQ for 6 days. (b) Viability is presented as percentage of ATP content of Mero-82 spheroids treated as described in (a). Data are presented as means +/- SD from ≥3 independent experiments. Significance was determined by Anova test.
