# Supplementary figures and images for: Inhibition of autophagy sensitizes malignant pleural mesothelioma cells to dual PI3K/mTOR inhibitors
Source: Cell Death Dis. 2015 May 7;6(5):e1757–. doi: 10.1038/cddis.2015.124 (PMC4669703; doi:10.1038/cddis.2015.124)

Supplementary Figure 1

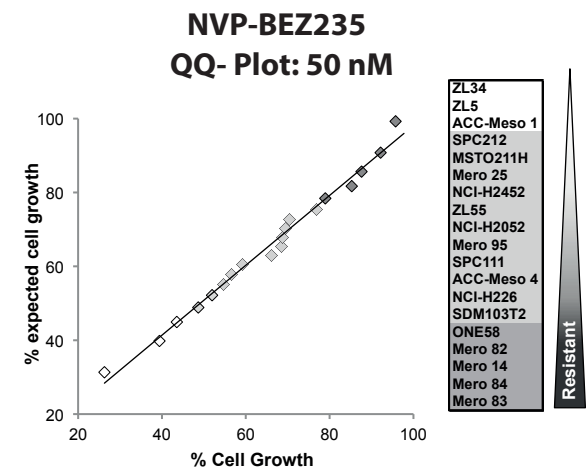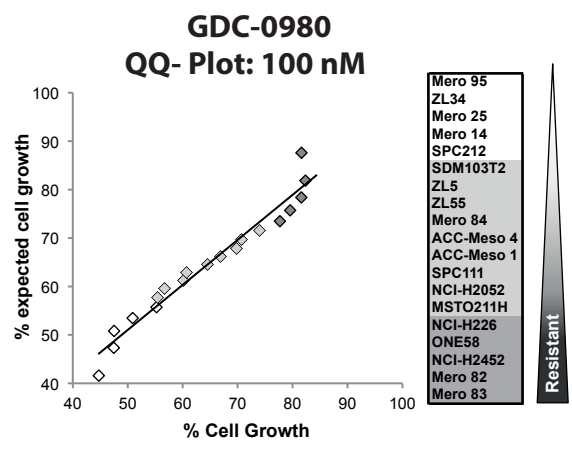

Supplement: Supplementary Figure 1 [file cddis2015124x2.pdf]

Supplementary Figure 2

72 h

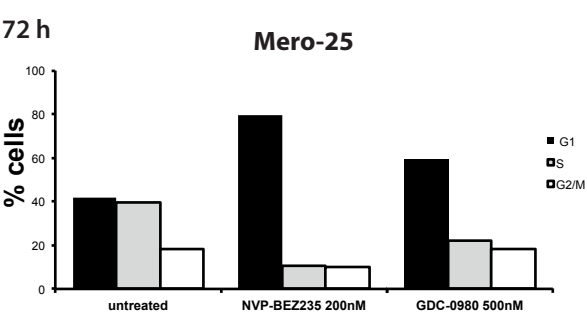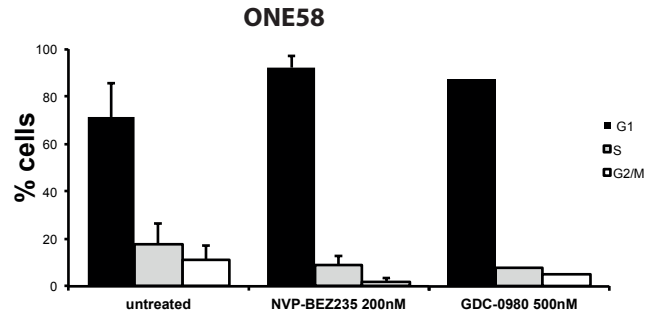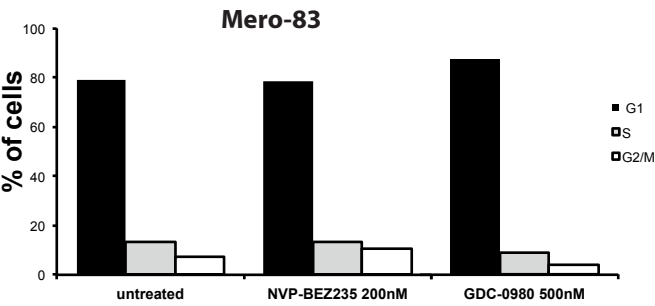

Supplement: Supplementary Figure 2 [file cddis2015124x3.pdf]

Supplementary Figure 3

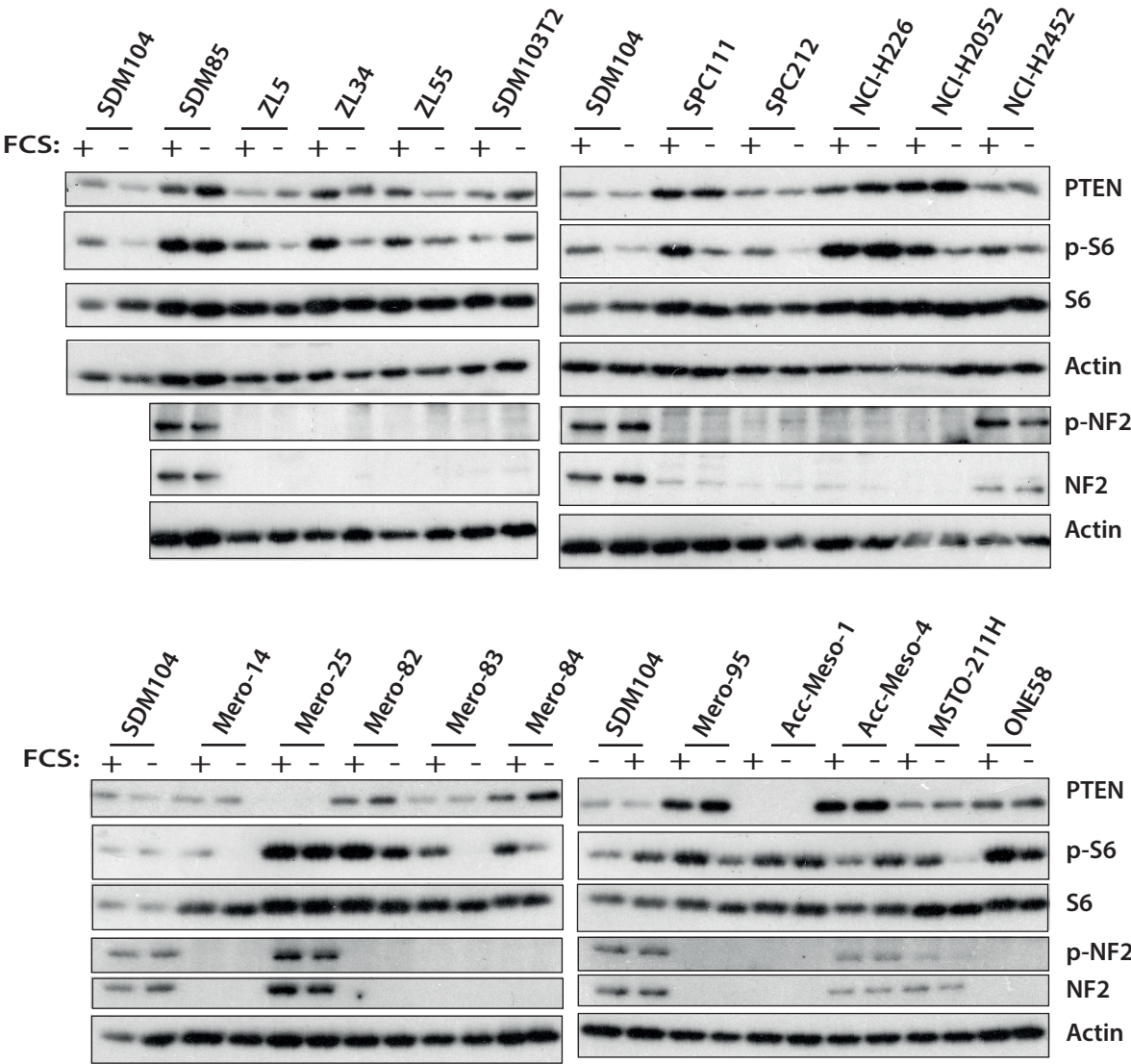

Supplement: Supplementary Figure 3 [file cddis2015124x4.pdf]

Supplementary Figure 4

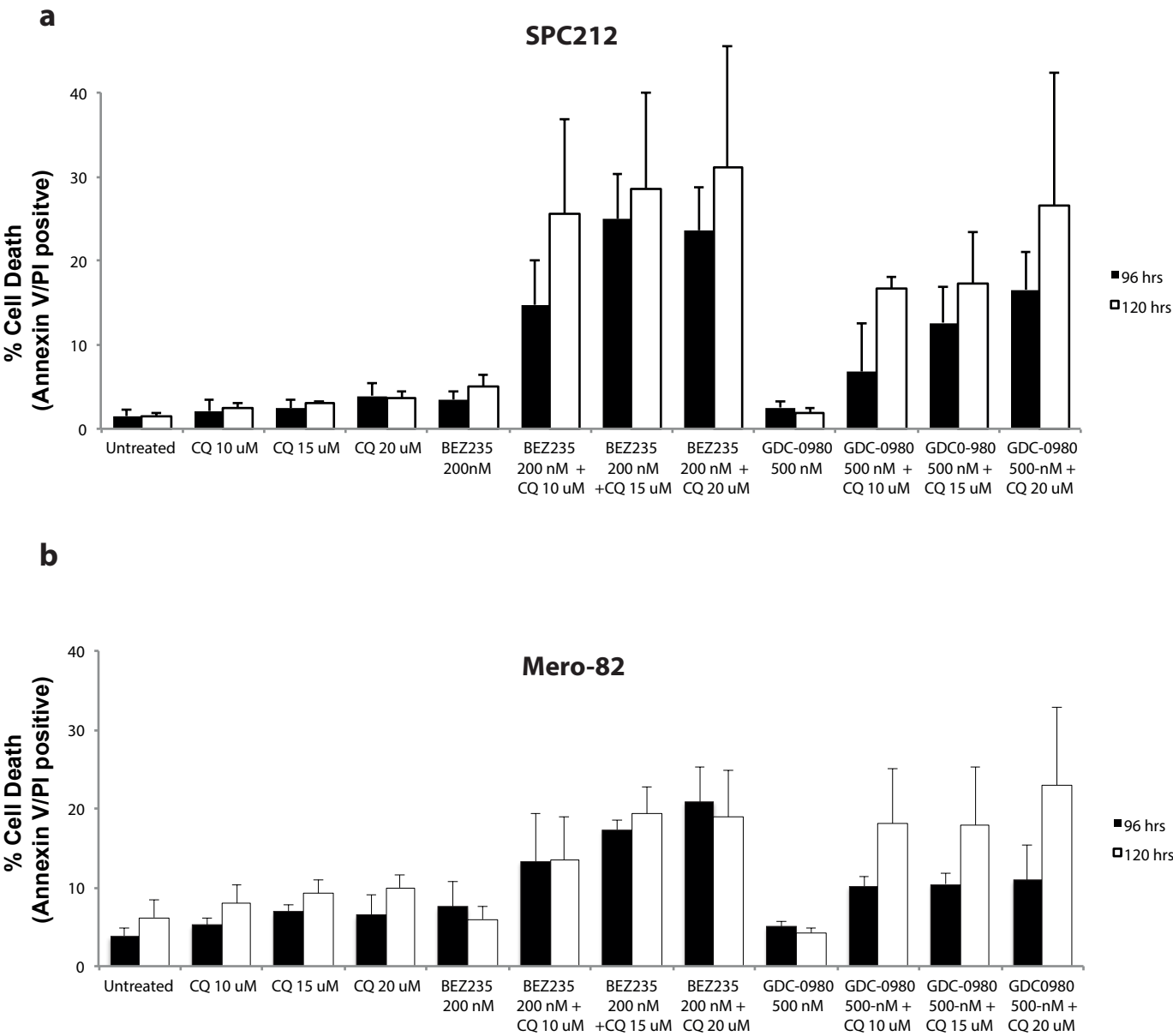

Supplement: Supplementary Figure 4 [file cddis2015124x5.pdf]

**Supplementary Figure 5**

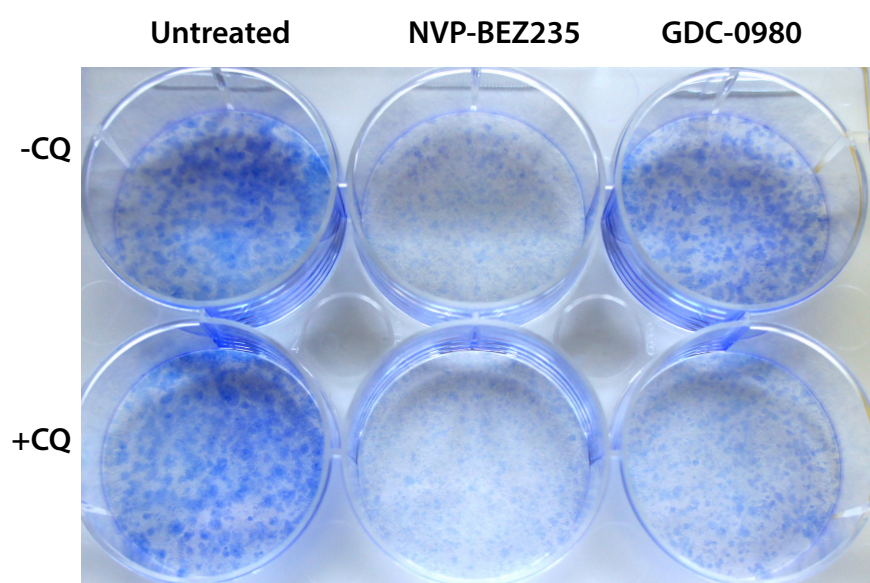

Supplement: Supplementary Figure 5 [file cddis2015124x6.pdf]

# Supplementary Figure 6

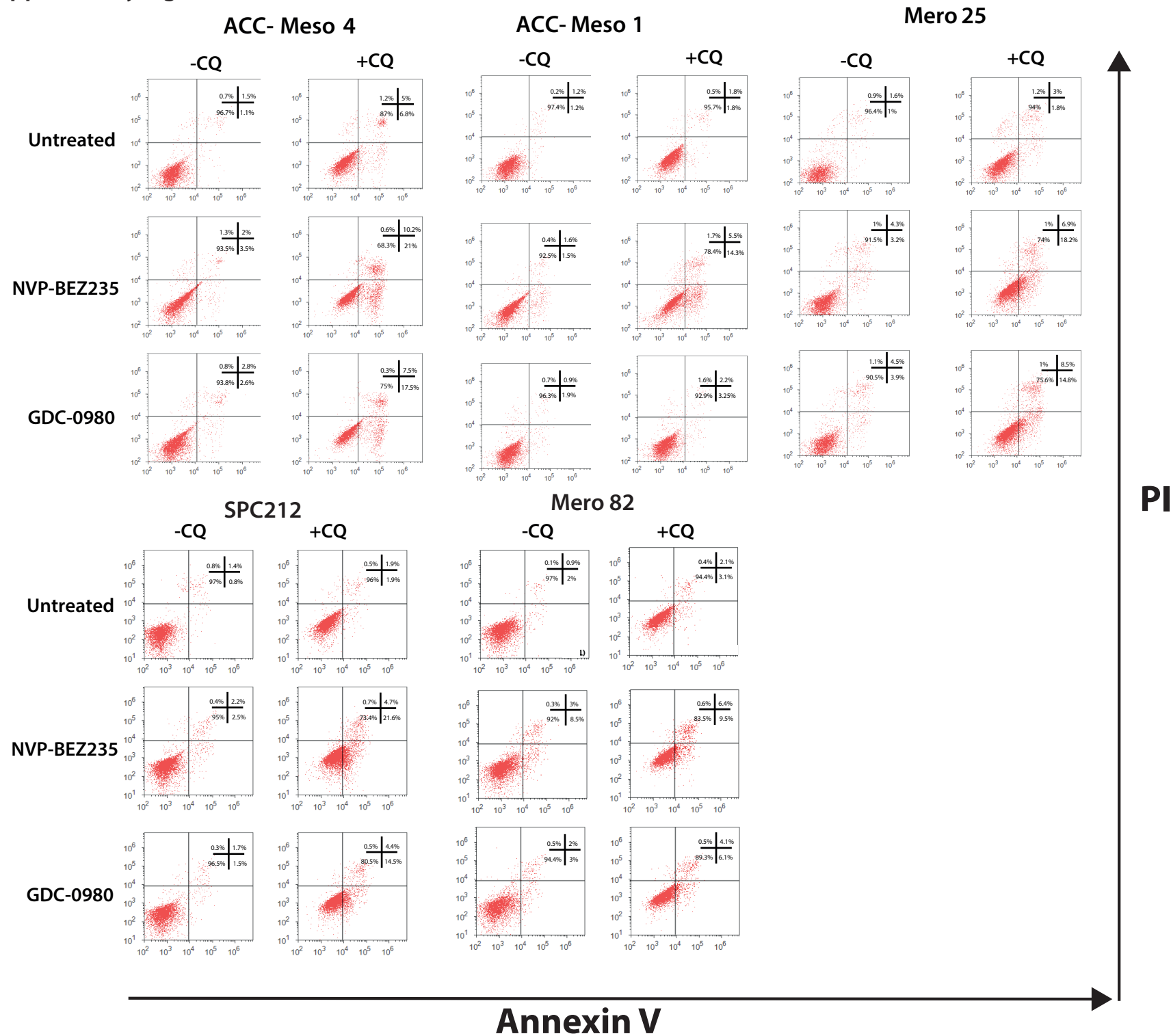

Supplement: Supplementary Figure 6 [file cddis2015124x7.pdf]

Supplementary Figure 7

96 h: Cytochrome c/ DAPI Staining

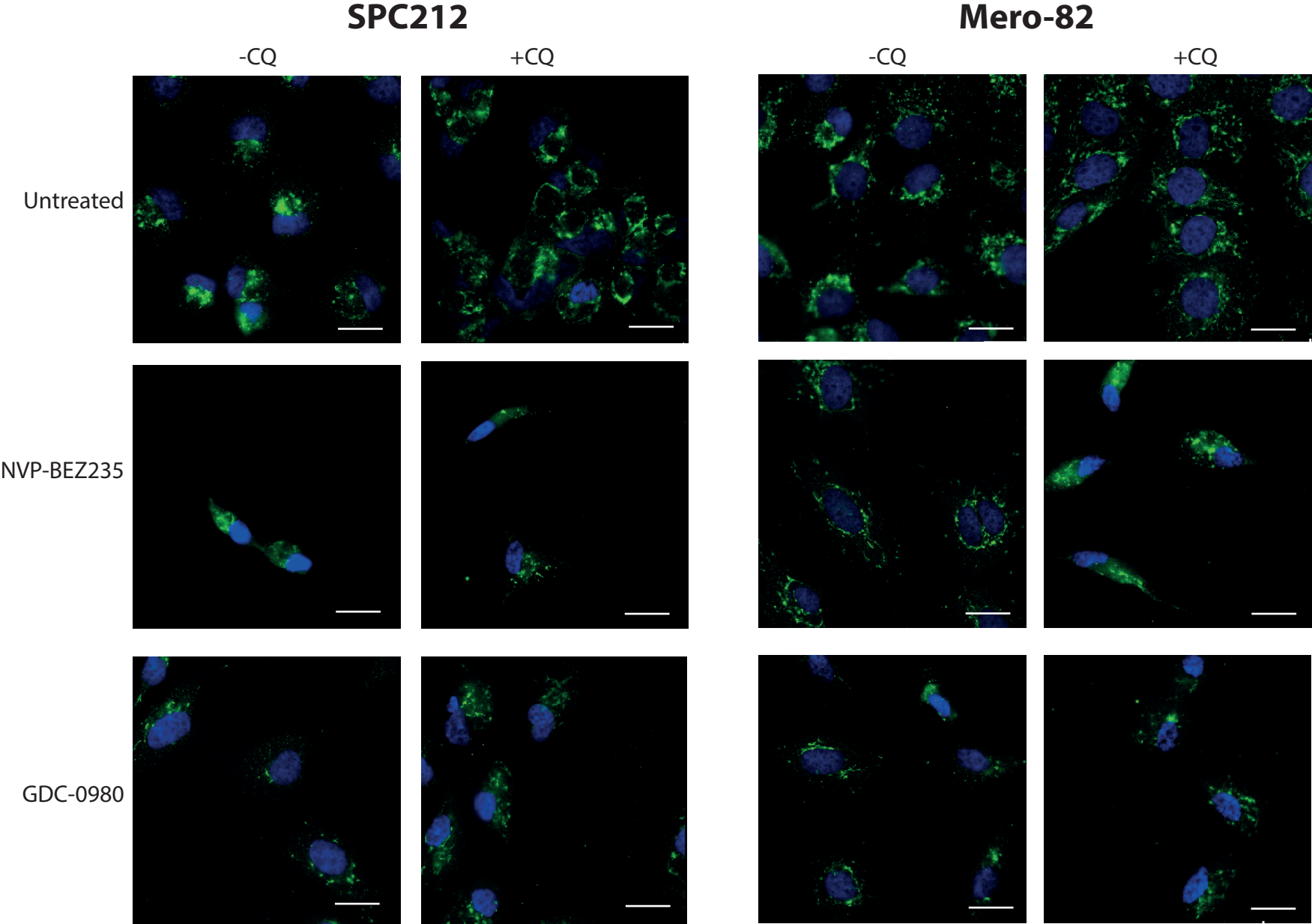

Scale Bar: 25 um

Supplement: Supplementary Figure 7 [file cddis2015124x8.pdf]

Supplementary Figure 9

SDM104

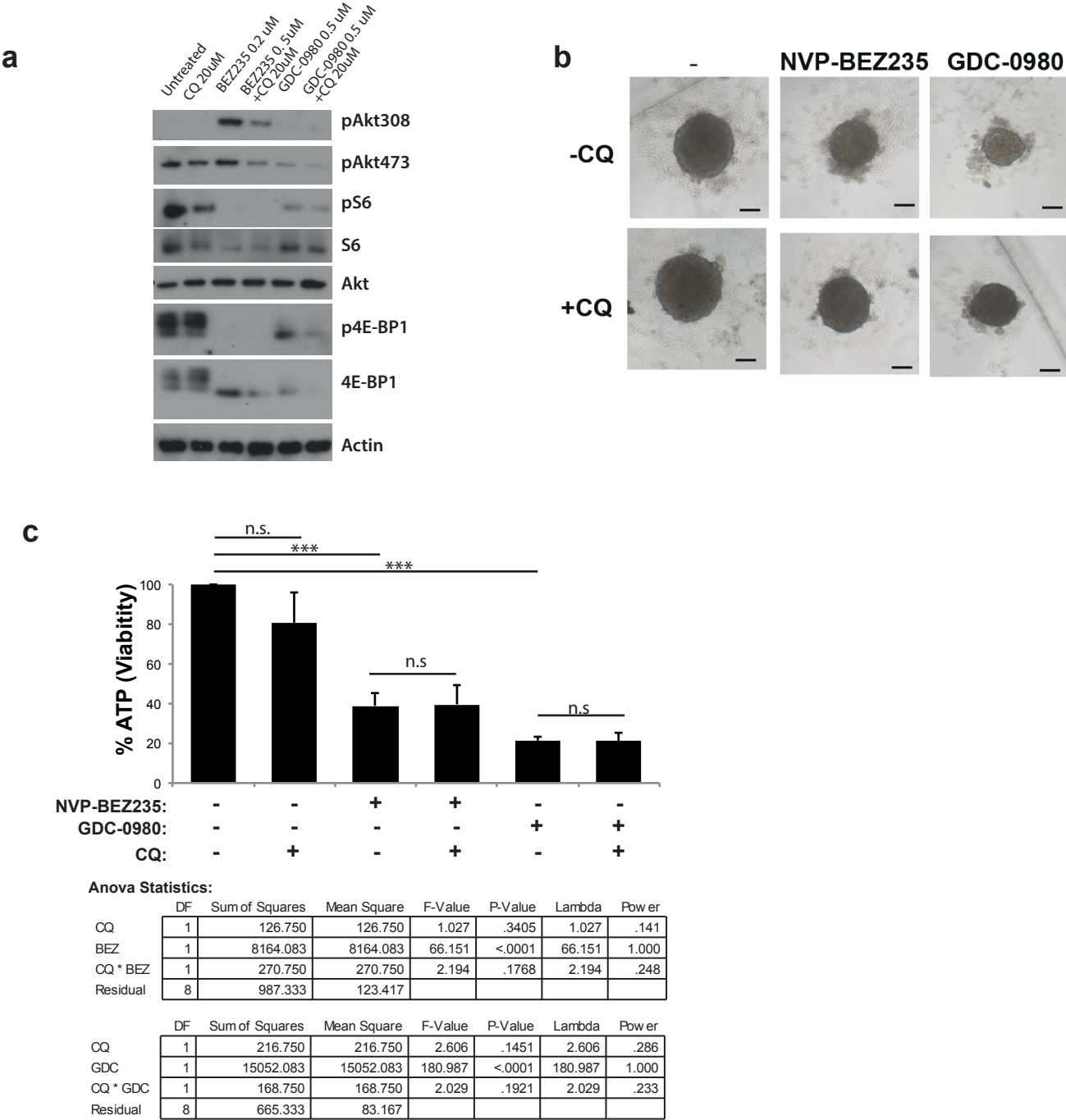

Supplement: Supplementary Figure 9 [file cddis2015124x10.pdf]
