# Supplementary material for: Inhibition of autophagy sensitizes malignant pleural mesothelioma cells to dual PI3K/mTOR inhibitors
Source: Cell Death Dis. 2015 May 7;6(5):e1757–. doi: 10.1038/cddis.2015.124 (PMC4669703; doi:10.1038/cddis.2015.124)
Supplement: Supplementary Figure 8 [file cddis2015124x9.pdf]

Supplementary Figure 8

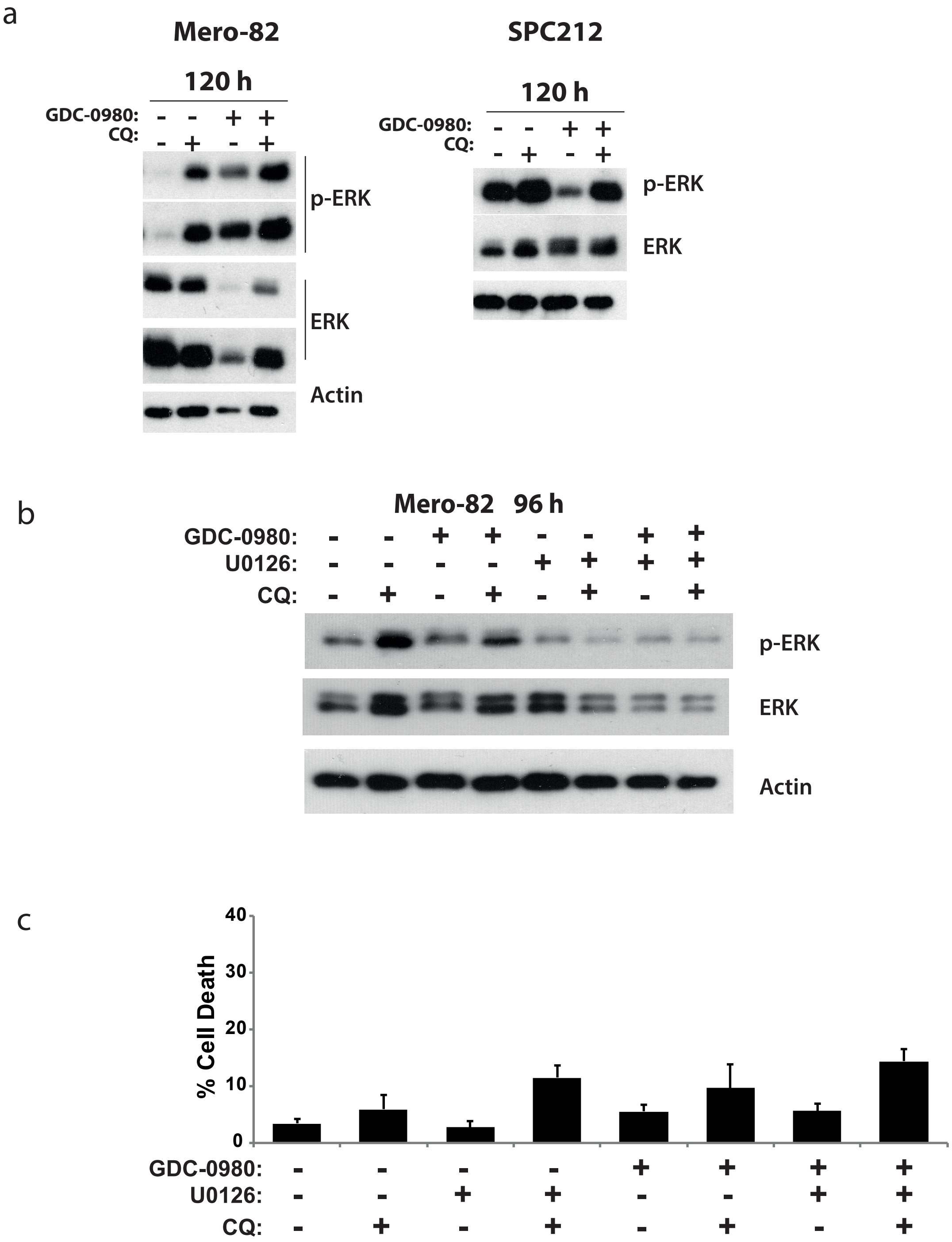

ANOVA Table for Column 1

	DF	Sum of Squares	Mean Square	F-Value	P-Value	Lambda	Power
CQ	1	308.960	308.960	48.515	<.0001	48.515	1.000
GDC	1	80.063	80.063	12.572	.0017	12.572	.940
U0126	1	31.173	31.173	4.895	.0371	4.895	.556
CQ * GDC	1	1.181	1.181	.185	.6708	.185	.069
CQ * U0126	1	39.883	39.883	6.263	.0199	6.263	.670
GDC * U0126	1	1.430	1.430	.225	.6401	.225	.073
CQ * GDC * U0126	1	1.132	1.132	.178	.6772	.178	.068
Residual	23	146.473	6.368				
