# Supplementary material for: Inhibition of autophagy sensitizes malignant pleural mesothelioma cells to dual PI3K/mTOR inhibitors
Source: Cell Death Dis. 2015 May 7;6(5):e1757–. doi: 10.1038/cddis.2015.124 (PMC4669703; doi:10.1038/cddis.2015.124)
Supplement: Supplementary Figure 10 [file cddis2015124x11.pdf]

Supplementary Figure 10

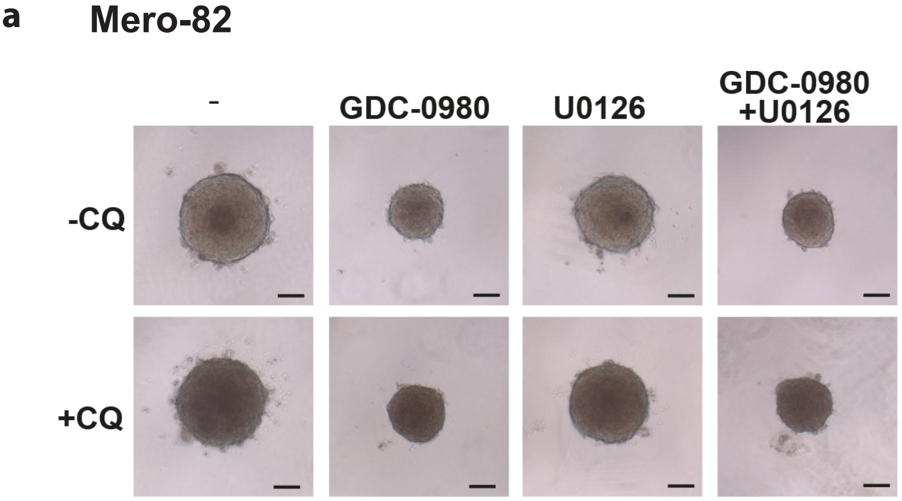

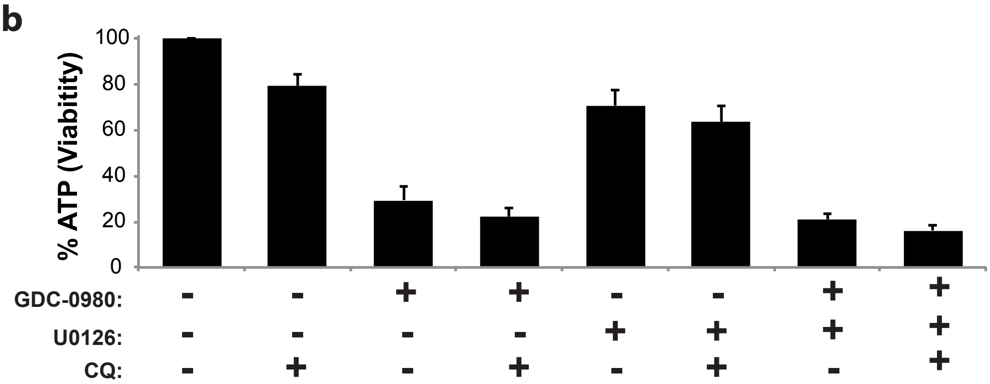

Anova Statistis:

	DF	Sum of Squares	Mean Square	F-Value	P-Value	Lambda	Pow er
CQ	1	663.441	663.441	32.270	<.0001	32.270	1.000
GDC	1	20915.773	20915.773	1017.356	<.0001	1017.356	1.000
U0126	1	1467.587	1467.587	71.384	<.0001	71.384	1.000
CQ * GDC	1	102.166	102.166	4.969	.0381	4.969	.555
CQ * U0126	1	115.904	115.904	5.638	.0283	5.638	.612
GDC * U0126	1	364.038	364.038	17.707	.0005	17.707	.987
CQ * GDC * U0126	1	55.183	55.183	2.684	.1178	2.684	.329
Residual	19	390.620	20.559				
